# Defect Detection of GFRP Composites through Long Pulse Thermography Using an Uncooled Microbolometer Infrared Camera

**DOI:** 10.3390/s24165225

**Published:** 2024-08-12

**Authors:** Murniwati Anwar, Faizal Mustapha, Mohd Na’im Abdullah, Mazli Mustapha, Nabihah Sallih, Azlan Ahmad, Siti Zubaidah Mat Daud

**Affiliations:** 1 Department of Mechanical Engineering, University Kuala Lumpur Malaysia France Institute (UniKL-MFI), Bandar Baru Bangi 43650, Malaysia; murniwati@unikl.edu.my; 2 Department of Aerospace Engineering, Faculty of Engineering, Universiti Putra Malaysia, Serdang 43400, Malaysia; naimabdullah@upm.edu.my; 3 Department of Mechanical Engineering, Universiti Teknologi Petronas, Seri Iskandar 32610, Malaysia; nabihah.sallih@utp.edu.my (N.S.); azlan.ahmad@utp.edu.my (A.A.); 4 LANL-JBNU Engineering Institute-Korea, Jeonbuk National University, 567 Baekje-daero, Deokjin-gu, Jeonju-si 54896, Jeollabuk-do, Republic of Korea; zubaidah.daud@jbnu.ac.kr

**Keywords:** defect, impact, IR camera, long pulse thermography (LPT), uncooled microbolometer, GFRP

## Abstract

The detection of impact and depth defects in Glass Fiber Reinforced Polymer (GFRP) composites has been extensively studied to develop effective, reliable, and cost-efficient assessment methods through various Non-Destructive Testing (NDT) techniques. Challenges in detecting these defects arise from varying responses based on the geometrical shape, thickness, and defect types. Long Pulse Thermography (LPT), utilizing an uncooled microbolometer and a low-resolution infrared (IR) camera, presents a promising solution for detecting both depth and impact defects in GFRP materials with a single setup and minimal tools at an economical cost. Despite its potential, the application of LPT has been limited due to susceptibility to noise from environmental radiation and reflections, leading to blurry images. This study focuses on optimizing LPT parameters to achieve accurate defect detection. Specifically, we investigated 11 flat-bottom hole (FBH) depth defects and impact defects ranging from 8 J to 15 J in GFRP materials. The key parameters examined include the environmental temperature, background reflection, background color reflection, and surface emissivity. Additionally, we employed image processing techniques to classify composite defects and automatically highlight defective areas. The Tanimoto Criterion (TC) was used to evaluate the accuracy of LPT both for raw images and post-processed images. The results demonstrate that through parameter optimization, the depth defects in GFRP materials were successfully detected. The TC success rate reached 0.91 for detecting FBH depth defects in raw images, which improved significantly after post-processing using Canny edge detection and Hough circle detection algorithms. This study underscores the potential of optimized LPT as a cost-effective and reliable method for detecting defects in GFRP composites.

## 1. Introduction

GFRP and its hybrid composite have been used in various application due to the thermal to mechanical qualities, such as the low weight, high specific strength and stiffness, corrosion resistance, good thermal insulation, fire-resistance, and thermodynamic stability [1,2,3,4]. Glass fiber reinforced polymers (GFRP) are being increasingly employed in many areas such as the aerospace, marine, civil application, electrical power generation, and transportation sectors [5,6,7,8,9,10,11]. Therefore, the assessment of structural health and identification of flaws and anomalies in the early stage is important to avoiding greater damage and catastrophe. The impact defects and depth defects of GFRP material were among the most common defects during manufacturing, and in-service defects have been studied using various NDT methods [12,13,14,15,16,17,18].

Thermography is a promising NDT technique for detecting composite defects in industrial systems due to its advantages such as being a non-contact method, providing real-time measurement, allowing for one-sided inspection, and featuring quick setup and integration capabilities [19,20]. Studies on thermographic NDT methods, including Pulse Thermography (PT), Long Pulse Thermography (LPT), Lock-in Thermography (LIT), and Step Heating Thermography (SHT), have demonstrated their effectiveness in detecting and characterizing composite defects [21,22,23,24]. Furthermore, integrating thermography with advanced processing techniques such as image processing and artificial intelligence enhances defect detectability and facilitates automatic defect detection and classification [25,26,27,28,29].

Among these methods, LPT stands out for its ability to identify deeper defects beneath the surface of GFRP composite materials using minimal and less sophisticated equipment, making it a cost-effective solution. This aligns with industrial needs to produce reliable and affordable monitoring systems, especially for small- and medium-sized enterprises [30,31,32]. However, the efficiency of the LPT method can be compromised by non-uniform heating and noise from different radiation sources, including the environment and background reflections. Additionally, the results can vary depending on the type of defect, material, and equipment used during measurement. Most research utilizes high-resolution IR cameras with cooling systems, which are less cost-effective. With the development of uncooled microbolometers and low-resolution IR cameras, which are more economical, it is possible to implement a cost-effective and reliable thermographic NDT method for assessing the internal health of composite materials.

**Figure 1:**
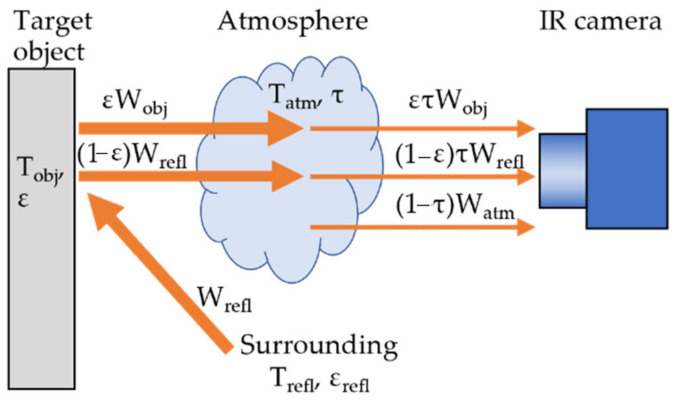
Radiation captured during data measurement using an IR camera [33].

During thermographic testing, the total radiation received by the IR camera comprises radiation from the sample, reflections, and atmospheric contributions. Surface material emissivity significantly influences the accuracy of GFRP defect detection. Figure 1 illustrates temperature measurements using an IR camera [33]. To minimize noise and enhance data accuracy, it is essential to control parameters such as the atmospheric conditions, environmental temperature, weather, and background reflections [34,35]. Various studies have investigated the impact of environmental conditions, such as ambient temperature and weather (sunny, rainy, and windy), on the assessment of construction material defects [36]. Indoor background reflections can stem from furniture, wallpaper, uneven painting, walls, surface roughness, and other factors, including wall color [34,37,38]. High background reflections can falsely elevate the temperature of the material being tested, leading to erroneous defect identification. Improvements for reducing background reflection include using polarizers attached to IR cameras [39] or specific post-processing algorithms [40,41]. Additionally, closed chambers or apparatuses can be employed to minimize reflection [42,43,44]. While standards and guidelines for thermographic NDT methods for detecting delamination in buildings and bridge concrete structures exist [35,45,46,47], guidelines for using LPT thermography with uncooled, low-resolution IR cameras to assess composite defects have yet to be established.

IR cameras are categorized based on the type of IR detector used, which is crucial for identifying and measuring infrared radiation. These detectors can be cooled or uncooled. Generally, cooled detectors in IR cameras for long-wavelength and mid-wavelength infrared (LWIR and MWIR) spectra are made of materials like Indium Antimonide (InSb) or Mercury Cadmium Telluride (MCT), offering better spatial resolution and a lower Noise Equivalent Temperature Difference (NETD), around 25 mK, compared to uncooled cameras, with NETD values of 40 mK or higher [33]. Despite their higher cost, uncooled IR cameras are more reasonable and advantageous due to their lighter weight and compact size, making them suitable for detecting defects in geometrically complex composites [48,49]. This factor has led to their application for surveillance systems in various fields such as in military applications [50]. However, limited studies have focused on using LPT with uncooled, low-resolution IR cameras to detect GFRP composites.

Numerous parameter optimizations have been proposed for thermographic methods to assess composite materials, including increasing the number of heating sources [44], improving the distance and angle position of the heating source relative to the sample [51], and extending the heating duration [22]. Various stimulation types have also been employed, such as halogen lamps [51,52,53], Xenon flash for line-scanning applications [52], and high-power LEDs [23]. However, improvements vary based on the application, material tested, and thermographic method applied.

Manufacturing defects like voids, porosity, and bubbles in composites are often simulated using flat-bottom hole (FBH) depth defects. Studies on FBH defects have been conducted with varying sizes and depths. After heating or cooling, heat dissipates through the composite material, revealing thermal discontinuities at defective sites. The challenge of detecting FBH defects lies in the temperature gradient affected by factors such as uneven heating and emissivity [54]. Previous research has compared SHT and PT methods, finding that defects at a 2 mm depth detectable for SHT and 1mm of PT [44]. Wang et al. also detected 2 mm depth defects at a 0.2 depth/diameter ratio using SHT and LPT methods [44]. Panela et al. applied various post-processing techniques to raw FBH defect images, identifying defects with depths ranging from 1.4 to 6.6 mm and diameters of 8 to 20 mm, achieving up to 60% accuracy using the Tanimoto criterion [55].

Simulated impact defects in composite materials replicate real-life damage scenarios such as bird strikes, collisions, and tool drops [56]. This event happened during maintenance and service phase [57]. If undetected early, impact defects can lead to severe damage, including fiber breakage, fiber shear-out, and matrix cracking [58], some of which are not fully understood [59]. Ongoing research on the mechanical characteristics of composite materials using thermographic NDT methods aims to improve defect assessment [60,61,62,63]. Despite extensive research, no studies have investigated the detection of GFRP impact and depth defects using the same configuration and parameters with uncooled microbolometers and low-resolution IR cameras to assess composite structural health.

The aim of this paper is to detect GFRP impact and depth defects using an uncooled, low-resolution IR camera. The study involves analyzing several significant parameters that influence defect detection accuracy and identifying optimal values for the LPT setup. The paper also reports the post-processing of raw images captured during LPT measurements, employing image processing to automatically detect and highlight defects. The Tanimoto criterion is used to verify the capability of the LPT method with an uncooled microbolometer IR camera in detecting GFRP impact and depth defects.

## 2. Materials and Methods

### 2.1. Test Sample

**Figure 2:**
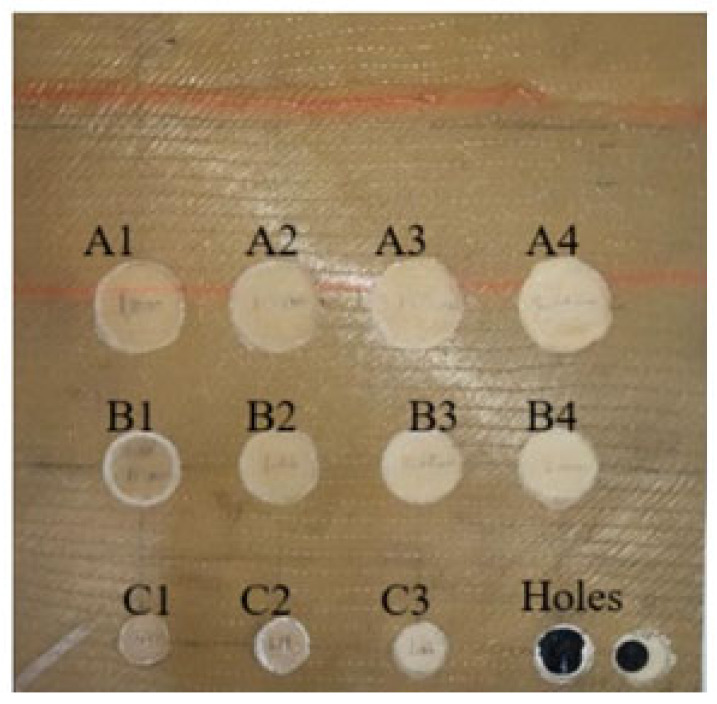
Front rear of the eleven flat bottom holes (FBH) of the GFRP sample.

**Figure 3:**
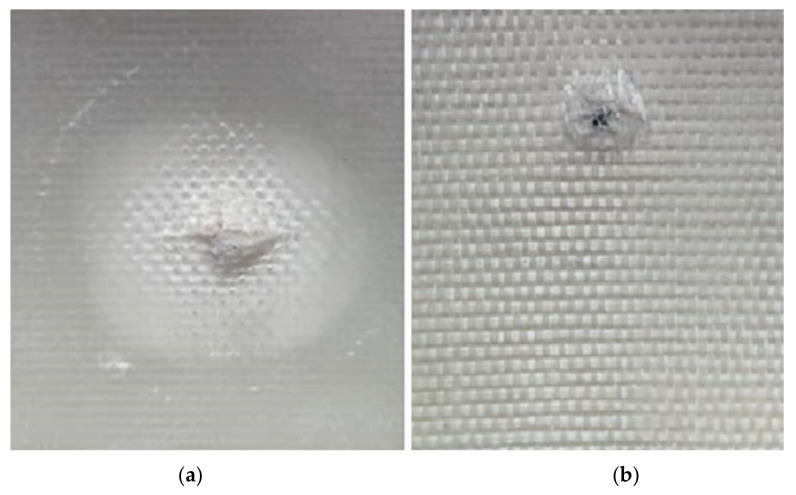
Impact defect of the GFRP sample: (**a**) sample IM1 and (**b**) sample IM2.

The test sample consists of a flat bottom hole sample A, made of a square-shaped plate of Glass Fiber Reinforced Polymer (GFRP) type C glass/Epoxy, 600 g/m^2^ with dimensions of 200 mm by 200 mm and a thickness of 3 mm. It is constructed from four layers of unidirectional roving of GFRP material. The plate includes eleven round-shaped, back-drilled flat bottom holes (FBH) with varying diameters and depths, as shown in Figure 2. Two small holes were intentionally created as references to distinguish between subsurface FBH defects and actual holes. The sizes and depths of each FBH defect are detailed in Table 1. Two medium-impact damage square shape samples, labeled IM1 and IM2 as shown in Figure 3, consisting of 14 layers of unidirectional roving with the dimensions of 150 mm × 150 mm at 6 mm of thickness, were also prepared using the same material. The impact energies were 8 J and 15 J, respectively. A striker, weighing 5.101 kg and featuring a hemispherical tip with a 5 mm radius, was used. The speed of the impact test is the gravitational acceleration = 9.81 m/s^2^. The magnitude of impact energy varied according to the height from which the striker was released on the sample, as described by Equation (1).

(1) $$E_{I}=mgh$$

### 2.2. Long Pulse Thermography (LPT) Configuration Setup

**Figure 4:**
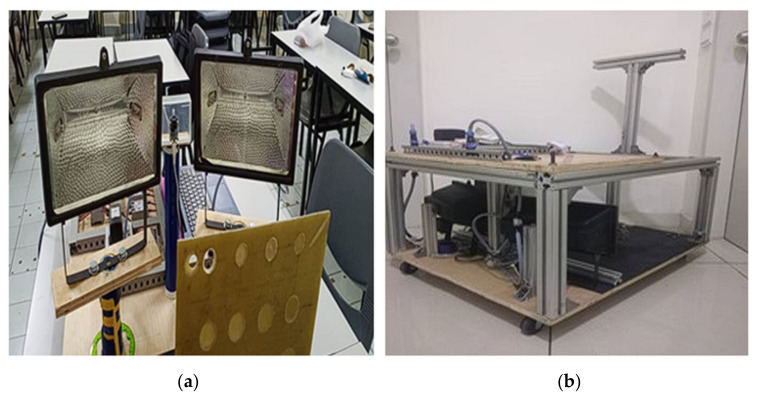
LPT setup using (**a**) reflex configurations and (**b**) an enclosure.

The experiment utilized a long pulse thermography (LPT) setup in a reflex configuration. Two sets of 1 kW halogen lamps served as the heating sources. The tests were conducted in both open and closed setups using an enclosure, as illustrated in Figure 4. In the reflex configuration, the camera and heat source were positioned on the same side, facing the sample, with each heating source angled between 25° and 30°. A flexible rail was attached to hold the sample, allowing it to be moved back and forth to adjust the heating distance between the heat source and the sample. The IR camera used in this research was an uncooled FLIR Lepton 3.5 IR camera, with an NETD of less than 50 mK and a resolution of 160 × 120. The IR camera detects wavelengths in the range of 8–14 μm.

### 2.3. Experiment Procedure on Selected Parameters Using LPT Thermography

From the literature, several important parameters that influence the detection of composite defects using the LPT method were identified. The optimized values for each parameter were determined during the experiments. These parameters are divided into four subcategories: material surface emissivity, internal enclosure background color reflection, background reflection, and environmental conditions and surrounding temperature, as shown in Table 2 [2,34,37,38].

#### 2.3.1. Environment Condition and Surrounding Temperature

LPT thermography testing was performed both indoors and outdoors at specific temperatures to evaluate the influence of environmental conditions. Indoors, thermal images were captured at room temperature (23 °C to 25 °C) and at a low temperature (16 °C to 18 °C) in an air-conditioned room. Outdoors, the test was conducted at 35 °C to 37 °C during sunny weather. The temperature was measured using a digital temperature and humidity meter (HTC-1) with an accuracy of ±1 °C and a range of −50 °C to +70 °C. During the time of the experiment, there was no detectable wind, as confirmed by observations. This controlled outdoor environment minimized the influence of external air movements on the thermal measurements. Heating durations ranged from 10 to 50 s, with images captured at 10 s intervals. During the experiment, 10 image frames were continuously captured, but only a few were recorded in the report to show the effect of temperature changes on the material.

#### 2.3.2. Parameter for Internal Enclosure Background Color Reflection

**Figure 5:**
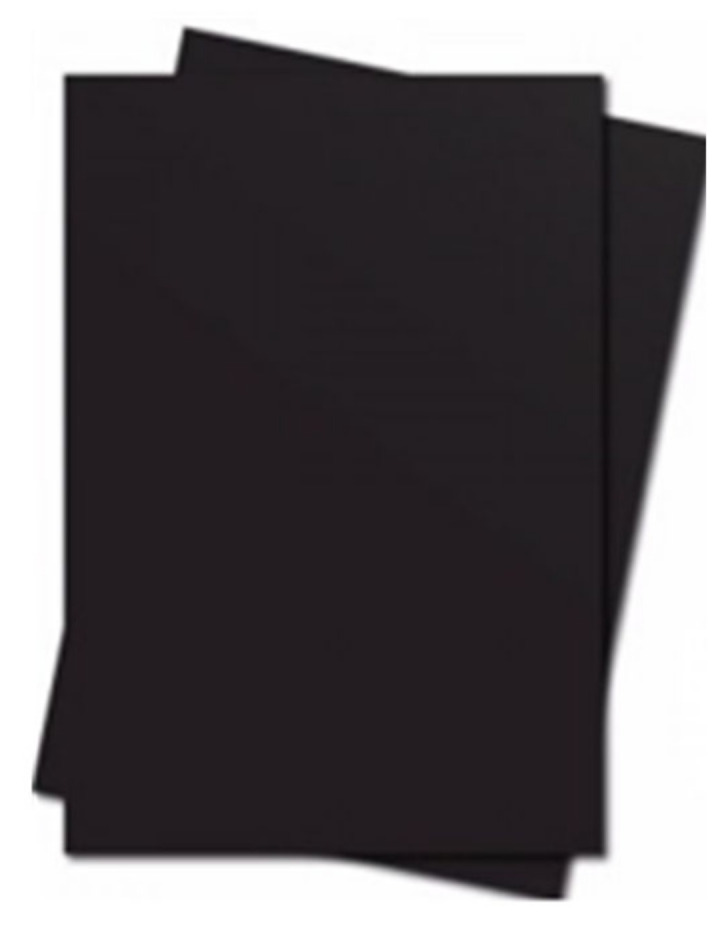
Example of black-colored paper used in the experiment.

Even though this research uses an enclosure to reduce the amount of external noise, it is important to minimize the background reflection that comes from the internal enclosure, especially with the application of an uncooled microbolometer IR camera that has a high NETD. A study showed that there were varying results in the application of different types of IR cameras for the detection of color painting defects [63]. Moreover, studies using a close setup are also being conducted using black color material, such as a black blanket [32] or a blackbody testing furnace [38]. This has shown that the internal background color of the enclosure influences the effectiveness of defect detection using a thermography system. The experiment was conducted by covering the internal walls of the enclosure with paper of three different colors: yellow, white, and black, with emissivity values of 0.68, 0.72, and 0.90, respectively (thermoworks.com/emissivity-table/ (accessed on 5 August 2024), as shown in Figure 5. The study was conducted indoors at a low temperature (16 °C to 18 °C).

#### 2.3.3. Parameter for Background Reflection

**Figure 6:**
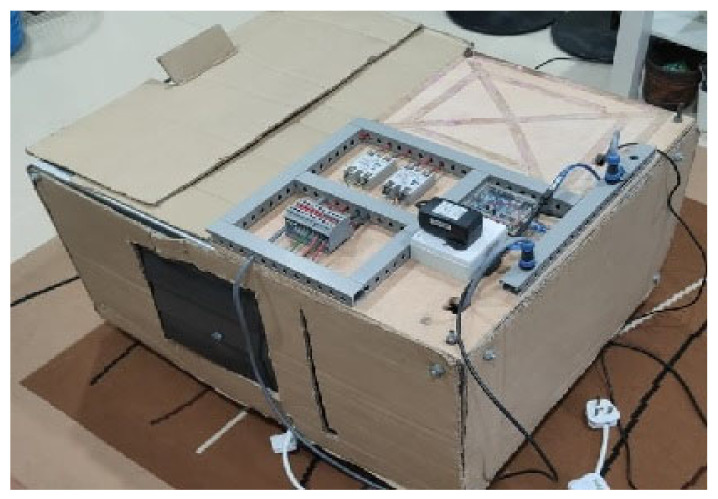
Close setup of the enclosure using hard cardboard.

Even though indoor applications did not receive direct sunlight, identifying background reflections was crucial. Background reflection sources could include furniture or walls where the test was performed [37,64]. The background reflection parameter was studied both with and without an enclosure, representing open and closed setups. The study was conducted indoors at a low temperature (16 °C to 18 °C). A closed setup was also tested using an enclosure made of hard cardboard, as shown in Figure 6.

#### 2.3.4. Material Surface Emissivity

**Figure 7:**
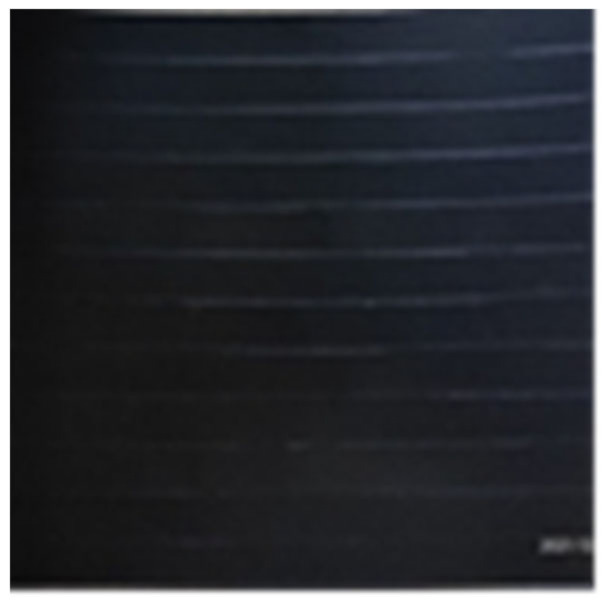
Surface material covered with color tape.

Most research covers the surface material with black paint to improve emissivity, but this method is not always practical. Therefore, emissivity investigation was performed by covering the GFRP material surface with black insulation tape (emissivity 0.97) [65]. A comparison test was also conducted without covering the surface, where the heat was applied directly to the GFRP material (emissivity 0.9) [20]. The experiment was conducted indoors using an enclosure, with the surrounding temperature controlled at a low temperature. Figure 7 shows how the surface material was covered.

### 2.4. Automatic Defect Detection Using Image Processing

This section explains the methodology for an automatic defect detection process aimed at highlighting defects while eliminating the background noise. Despite optimizing the parameters during the experiment, small flaws can still be obscured by noise and cannot be entirely eliminated [66]. Additionally, the low resolution of the IR camera (160 × 120 pixels) results in small and pixelated images. To address this, image-processing techniques were applied to enhance the captured images.

**Figure 8:**
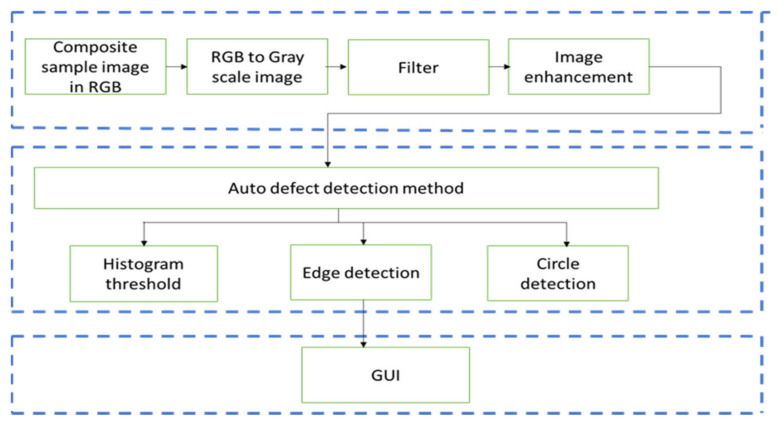
Image-segmentation method for automatic defect detection.

An automatic composite defect detection method was developed to enhance the defective areas and remove the surrounding noise. Raw images captured during the LPT thermography experiment were low-resolution and noisy, necessitating pre-processing to filter out the noise and enhance the image quality. Image processing was performed on individual grayscale images to reduce the computational time. The study proposed three image segmentation approaches, each exhibiting different features for automatically identifying FBH depth and impact defects in GFRP samples [67]. Automatic defect detection is crucial in industrial applications, as it enhances manufacturing efficiency by enabling quicker decisions on sample acceptance and rejection without relying on skilled workers to interpret raw images. Figure 8 shows the flowchart of the image-processing method applied in this study. The process flow is divided into three sections as shown in blue dotted boxes.

**Figure 9:**
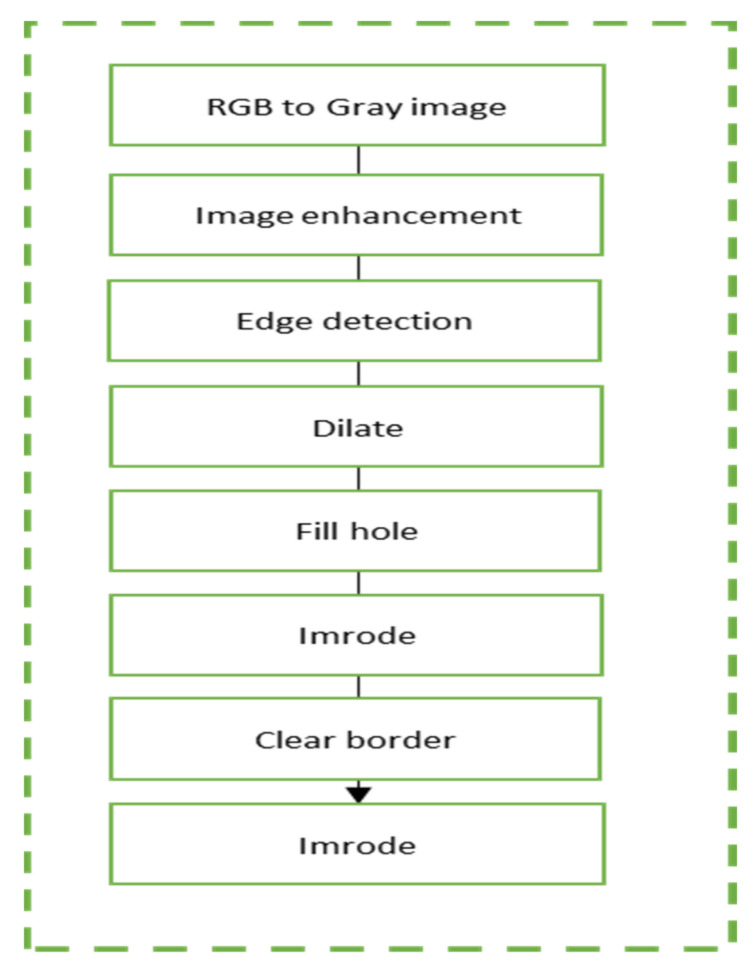
Edge detection flowchart.

Edge detection segmentation is an image algorithm used to identify points with strong edges in images. In this experiment, two traditional edge detection operators, Canny and Sobel, were implemented. Both operators have been used in numerous applications, including crack detection in metal pipes [68] and medical image processing [69]. Sobel operators consist of 3 × 3 convolution operations, with one being a 90° rotation of the other. Edge detection transforms the image from grayscale to binary, represented by 1 (white) and 0 (black). Morphological operations were utilized to highlight only the defective areas. The process began with dilation to expand the radius of the defective areas and connect any broken edges identified by the Canny or Sobel operator, followed by erosion to shrink the radius back to its initial size and remove noise. This process ensured that the image size remained constant before and after processing. Figure 9 shows the flowchart for detecting composite defects using edge detection segmentation.

**Figure 10:**
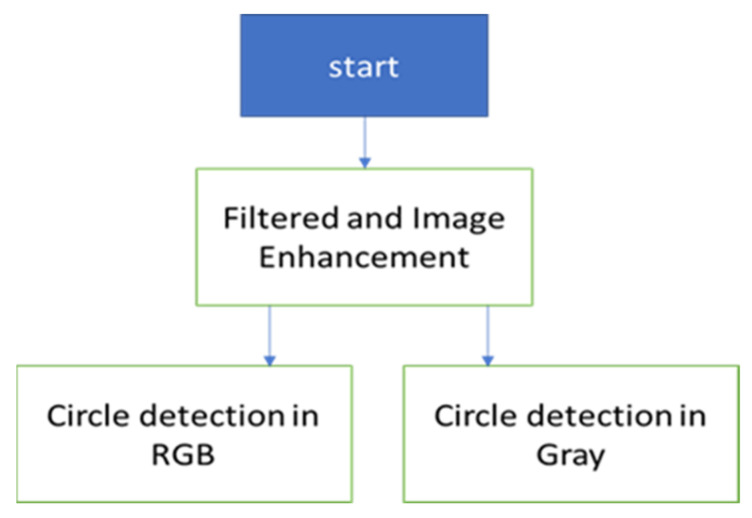
Circle detection algorithm flowchart.

Another promising segmentation method for automatic defect detection is histogram thresholding, a technique used in various fields such as brain tumor detection [70]. Identifying an optimal histogram threshold value is crucial to ensuring no defects are missed and no noise is included in the process. In this method, the optimal threshold for histogram segmentation was set at 200, resulting in a binary image where the defective areas are represented by white (logic 1). FBH defects typically represent manufacturing defects such as voids and bubbles, while impact defects arise from operational incidents such as collisions or impacts. FBH depth defects are usually round-shaped, while impact defects lack specific shapes. Therefore, in this research, a circle detection algorithm, known as the Circular Hough Transform (CHT) algorithm, was appropriate for detecting FBH depth defects. The algorithm was applied to both grayscale and RGB images, as shown in Figure 10.

The evaluation of the LPT method using an uncooled microbolometer and low-resolution IR camera on FBH composite defects in GFRP was performed using visual perception and the Tanimoto criterion (TC) [55,71]. This method measures the effectiveness of thermographic NDT before and after image processing in detecting flaws and anomalies. The Tanimoto criterion decision incorporates true positive (TP), true negative (TN), and false negative (FN) counts. The calculation for TC efficiency is given by Equation (2):

(2) $$TC=\frac{N_{rd-}N_{md}}{N_{rd+}N_{fd}}$$

where:

$$N_{rd}=numbers\;of\;real\;defect$$

$$N_{md}=numbers\;of\;missed\;defect$$

$$N_{fd}=numbers\;of\;false\;defect$$

## 3. Results

### 3.1. The Environmental Temperature Indoors and Outdoors

**Figure 11:**
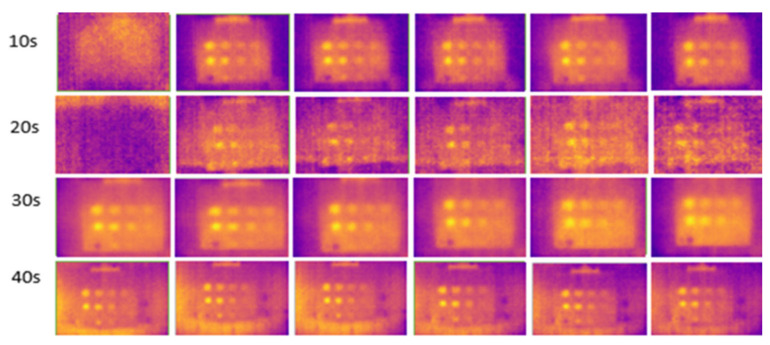
Outdoor output image at temperatures above 35 °C for 10–40 s of heating duration.

**Figure 12:**
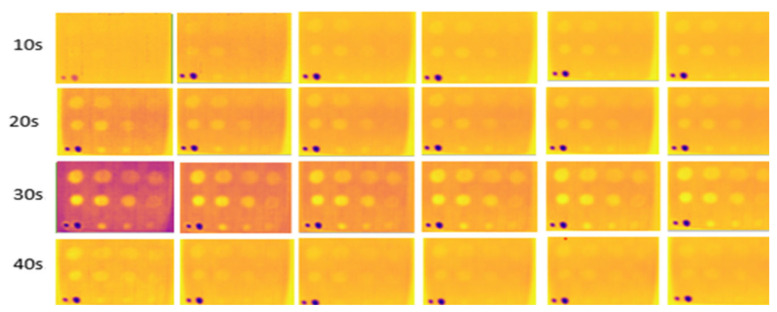
Indoor output image at room temperature (23–25 °C) for 10–40 s of heating duration.

**Figure 13:**
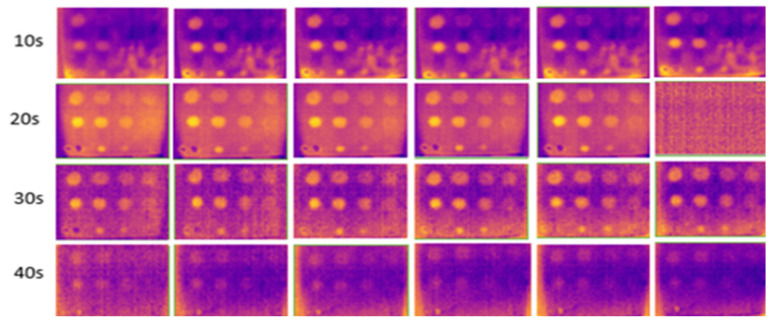
Indoor output image at low temperatures (16–18 °C) for 10–40 s of heating duration.

The results for outdoor temperatures from 35 °C to 37 °C are shown in Figure 11, compared with indoor temperatures from 23 °C to 25 °C (room temperature) and from 16 °C to 18 °C (low temperature in an air-conditioned room), as shown in Figure 12 and Figure 13, respectively.

**Figure 14:**
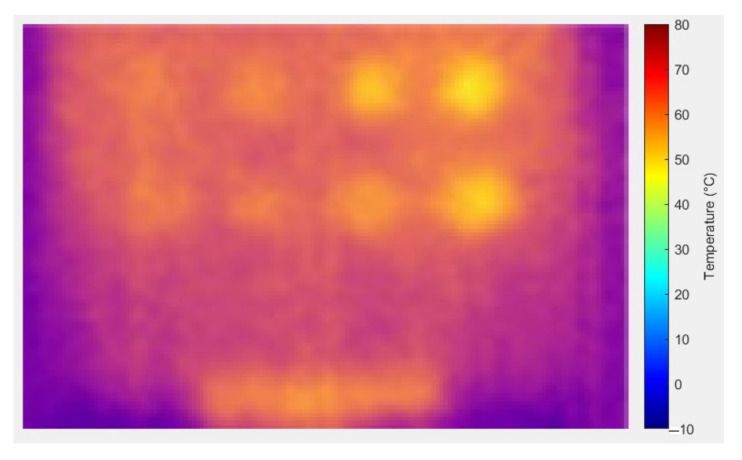
Temperature bar for one of the images captured outdoors at temperatures above 35 °C.

During the experiment, ten image frames were continuously captured, but only six images were recorded at the horizontal position at each heating period shown in each figure. These images were used to highlight the effect of temperature changes on the material. Figure 14 shows the temperature bar for one of the sample images captured outdoors at a temperature above 35 °C. The result showed that the temperature is not evenly distributed. The temperature in the defective areas varies from 45 °C to 55 °C. It is also demonstrated that the images captured outdoors were unclear and noisy. As the heating duration increased, the results remained constant, without any improvement. The experiment demonstrates that indoor tests produce less noise compared to outdoor tests due to controlled surrounding temperatures, eliminating radiation caused by external heat sources, particularly solar radiation. At 10 s of heating, the results at room temperature and air-conditioned room temperature showed very few FBH composite defects detected, indicating insufficient heating on the material’s surface to produce a significant temperature contrast between defective and non-defective areas. As the heating duration increased to 20 s, more defects were detected, achieving a 91% detection rate. The B1 defect was detected with low observability, while the C1 defect showed very low observability, making it undetectable. Both defects have the highest depth at 2.88 mm but have different diameters. At room temperature, similar detection results appeared but required longer heating durations (30 s). However, as the heating duration extended to 40 s, the defective areas on the samples became less evident and started to vanish because the surface temperature of the samples equalized between defective and non-defective areas.

### 3.2. Internal Enclosure Background Color Reflection

**Figure 15:**
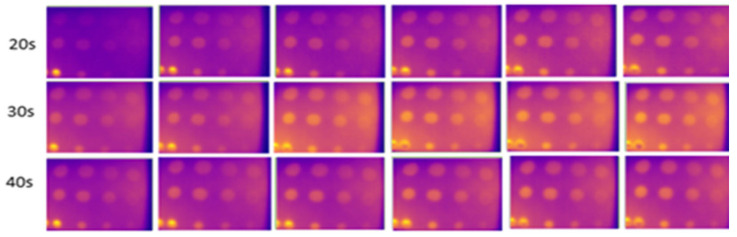
Output image for the black-colored background of the internal wall for 20–40 s of heating.

**Figure 16:**
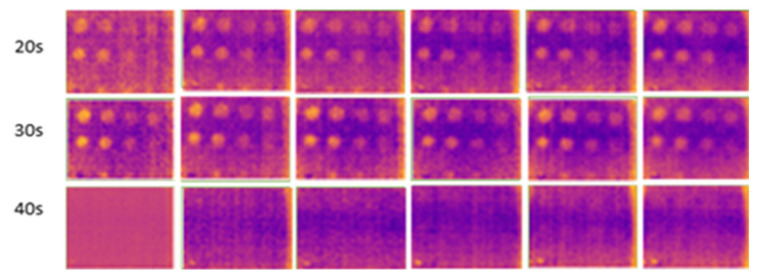
Output image for the white-colored background of the internal wall for 20–40 s of heating.

**Figure 17:**
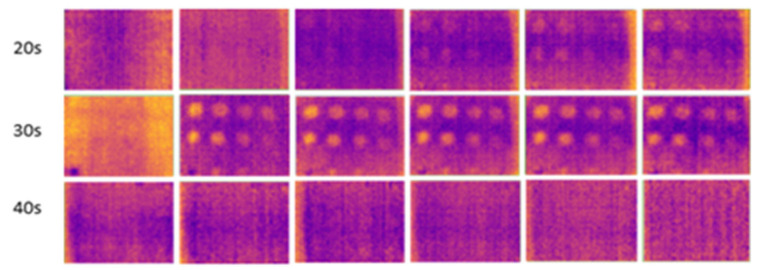
Output image for the yellow-colored background of the internal wall for 20–40 s of heating.

This experiment, conducted indoors at low temperatures (16 °C to 18 °C), focused on three different internal background colors of the enclosure. Heating durations were limited to 20 to 40 s, as this range adequately captured the thermal contrast between defective and non-defective areas. Images captured against a black background, as shown in Figure 15, had higher clarity, less noise, and more observable defects compared to those captured against white and yellow backgrounds, shown in Figure 16 and Figure 17. At 30 and 40 s of heating, the black background achieved optimal defect detection (91%). However, the B1 defect had poor visibility and the C1 defect was undetectable due to their size and depth. The heat absorption by the black background, with an emissivity close to 1, minimized reflection and noise, resulting in clearer defect detection.

### 3.3. Background Reflection Parameter

**Figure 18:**
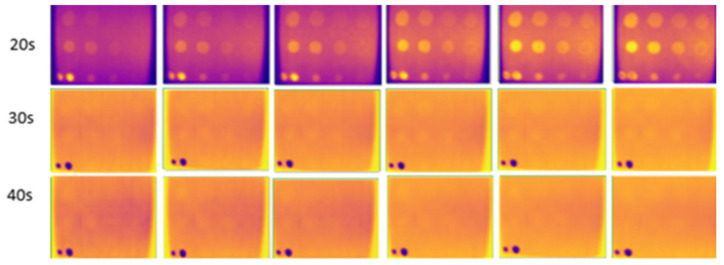
Result for indoors, without an enclosure at temperatures from 16 °C to 18 °C from 20 s (first row) to 40 s (last row) of heating.

**Figure 19:**
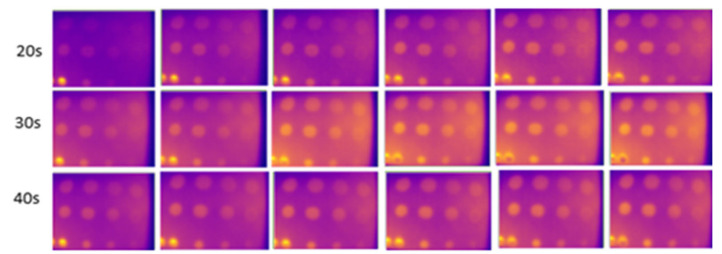
Result for indoors, using an enclosure at temperatures from 16 °C to 18 °C from 20 s (first row) to 40 s (last row) of heating.

Figure 18 and Figure 19 show the results of experiments conducted indoors at 16 °C to 18 °C with and without an enclosure. At 20 s of heating, ten defects were observable, with slight variations during the cooling periods under the two conditions. The C1 defect, with a depth-to-size ratio of 0.206, remained undetectable. This experiment demonstrates that using a closed setup, such as an enclosure, minimizes heat loss and reduces interference from external heat sources like furniture and walls. The findings support previous studies that have demonstrated that the efficiency of detection results will improve with the use of confined spaces [44,71] and black-colored background material [32] in enhancing defect detection.

### 3.4. Surface Emissivity Parameter

**Figure 20:**
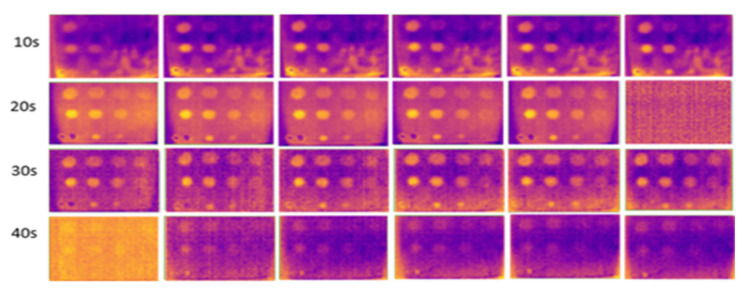
Without tape covered on the surface material at low temperatures (16° C to 18 °C) from 10 s (first row) to 40 s (last row) of heating.

**Figure 21:**
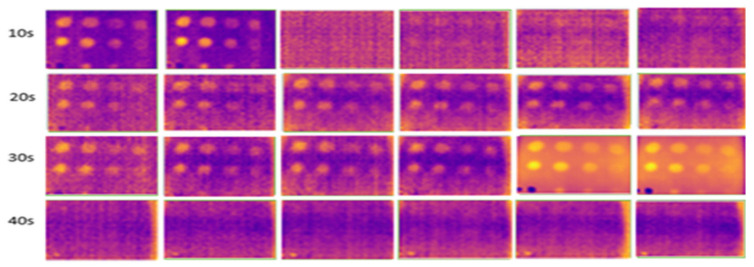
Yellow tape covered on top of the surface sample at low temperatures (16 °C to 18 °C) from 10 s (first row) to 40 s (last row) of heating.

**Figure 22:**
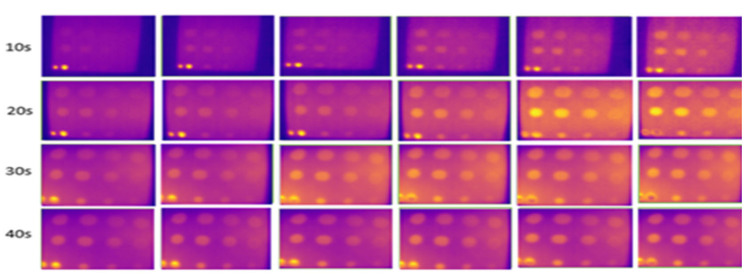
With black tape on the surface material at low temperatures (16 °C to 18 °C) from 10 s (first row) to 40 s (last row) of heating.

At 10 s of heating, the images captured with any surface emissivity were noisy, with fewer detected FBH defects, as shown in Figure 20, Figure 21 and Figure 22. This is due to insufficient heat application on the GFRP material surface. As the heating duration extended from 20 to 30 s, more defects were detected. Images captured without black insulation tape were less noisy and clearer, with heat evenly distributed compared to other surface emissivities. Black tape, with a higher emissivity value of 0.97 compared to the GFRP surface (0.9), improved detection. At 30 s of heating, 10 defects were detected with black tape, though the B1 defect had poor visibility and the C1 defect remained undetectable.

### 3.5. Optimized Parameter

The selected parameters were tested and analyzed using the LPT thermography setup with an uncooled, low-resolution microbolometer IR camera. The results indicated that the indoor configuration, operating at low temperatures (16 °C to 18 °C) and room temperatures (23 °C to 25 °C), performed well in terms of control and exhibited reduced noise compared to the outdoor setup at 35 °C to 37 °C during sunny days, which suffered from diverse uncontrollable background reflections from heat radiation sources. The effective heating duration and heating distance for a square GFRP plate, comprising 20 layers measuring 200 mm × 200 mm with a thickness of 3 mm, was at 20 cm and 25 cm, together with a heating duration ranging from 30 s to 40 s.

**Figure 23:**
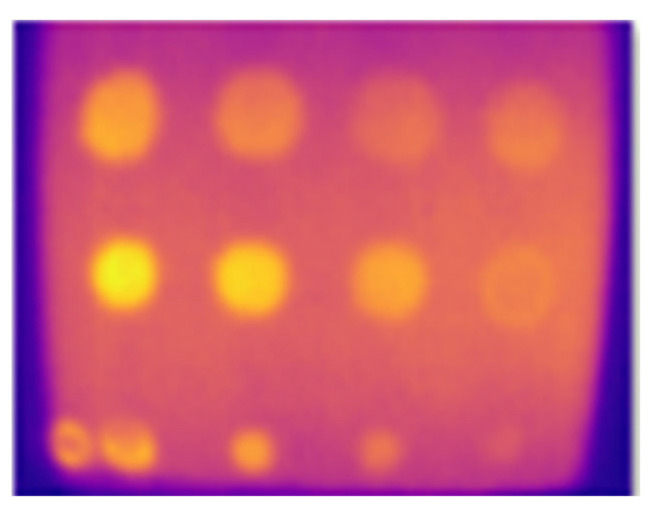
Optimized FBH defect of the GFRP detected.

The emissivity of the surface material was a crucial parameter in LPT. Surface GFRP typically exhibits a low emissivity value. Therefore, to enhance detection capabilities, applying black tape or black paint can improve the emissivity of the material. Figure 23 displays the results of the optimal values for each selected parameter. The analysis revealed that 10 defects were detected. However, defect B1, with a depth of 2.88 mm and a diameter of 21 mm, had low observability, while defect C1, with the same depth of 2.88 mm but a smaller diameter of 14 mm, had extremely low observability. Table 3 presents the optimal values for each evaluated parameter.

**Figure 24:**
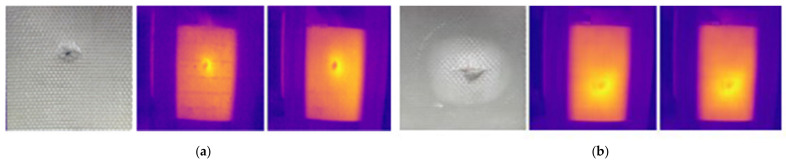
Impact defect detection results using optimized parameters for samples (**a**) B1 and (**b**) B2.

Figure 24 illustrates the assessment of samples B1 and B2 using optimized parameters using the LPT method. The impact defects on the composite material can result in matrix cracking and fiber breakage, as composites are anisotropic materials. The impact defects of samples B1 and B2 were clearly captured using the optimized parameters. Holes and damaged areas can also be seen in the images. However, detailed damage such as small crack sizes cannot be observed due to the low resolution of the IR camera used for capturing the images.

### 3.6. Image Processing Result

**Figure 25:**
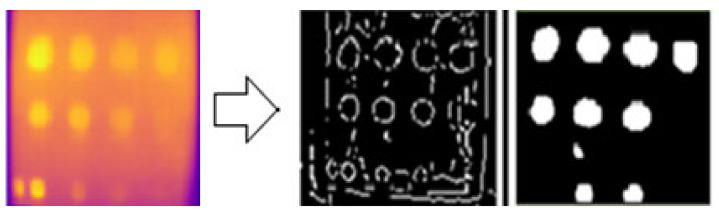
FBH depth defect detection process using Canny edge detection segmentation.

**Figure 26:**
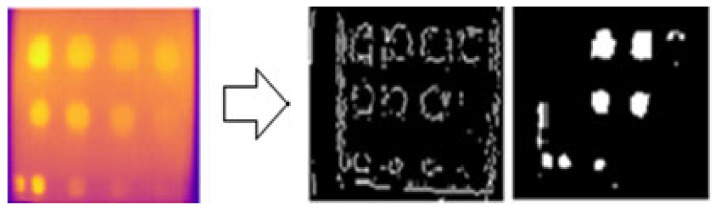
FBH depth defect detection process using Sobel edge detection segmentation.

The results of the edge segmentation method for FBH depth defects on sample A are presented in Figure 25 and Figure 26 for the Sobel and Canny operators, respectively. Several threshold values were tested to detect as many edge defect boundaries as possible. The Sobel operator at a threshold of 0.03 and the Canny operator at a threshold of 0.094 produced the optimal results. The Canny operator outperformed the Sobel operator, detecting nine defects compared to four with Sobel. However, some noise was mistakenly detected as defects in both operators.

**Figure 27:**
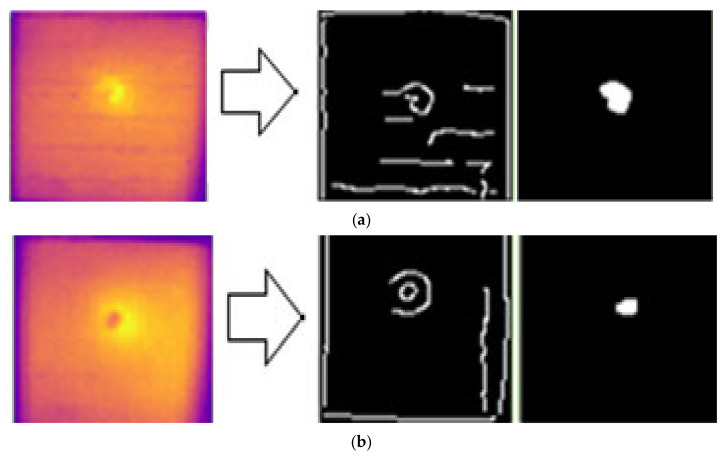
Edge image segmentation for GFRP impact defect detection using the Canny edge detection method for (**a**) defect IM1 and (**b**) defect IM2.

Figure 27 shows the output results of the Canny edge detection applied to the impact defects of the B1 and B2 samples. The results indicated that the edge detection method was able to detect the center of the impact defects. This method has the limitation of being unable to detect cracks and delamination surrounding the impact area. Additionally, for impact defects, the shape of the defect changed after applying the morphological process to the image.

**Figure 28:**
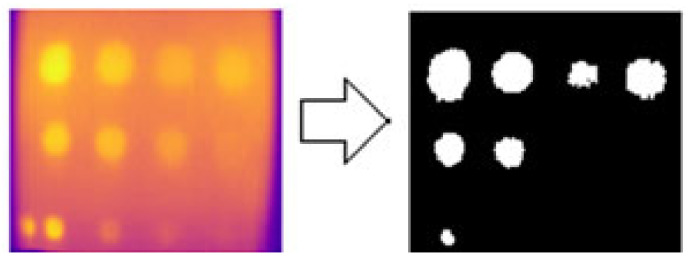
FBH depth defect detection using histogram threshold.

**Figure 29:**
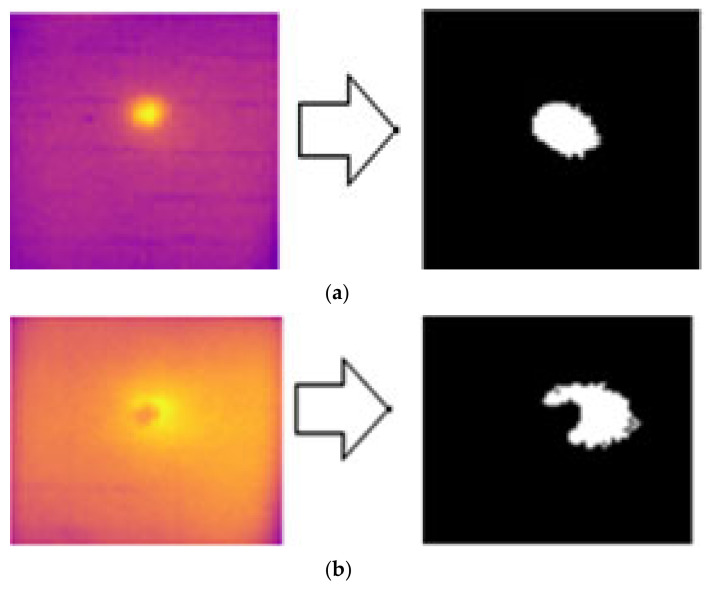
Histogram threshold segmentation method result for (**a**) defect IM1 and (**b**) defect IM2.

**Figure 30:**
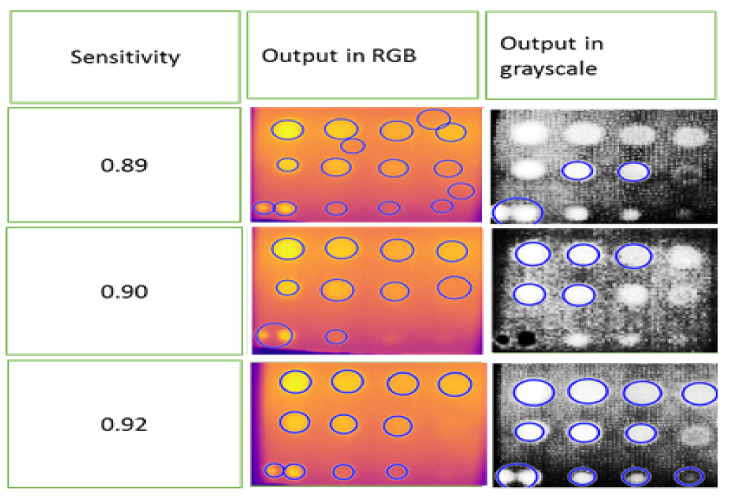
FBH defect detection using the circle segmentation method.

Another method of defect detection using image processing is histogram segmentation, which analyzes the histogram of an image and determines the pixel threshold value to distinguish between defective and non-defective areas. The optimal intensity threshold for histogram segmentation suitable for FBH depth and impact defects was found to be 200 pixels. However, using this method, only six defects were detected for FBH depth, including A1 to A4 defects and B3 and B4 defects, as shown in Figure 28. Moreover, the size of the detected defects appeared larger than the actual defect images due to heat distribution variability. The results for impact defects in Figure 29 and Figure 30 also showed a wider area of defect detection. This occurred because the histogram segmentation in this study was based on light intensity, influenced by heat distribution during the cooling period, which may have covered a larger area than the defective region.

The circle detection segmentation method is only suitable for FBH depth defects due to their geometry. In this study, only the FBH depth defect was utilized to test the circle identification method or Hough algorithm on grayscale and RGB color images. For this function, the pixel size and type of circle must be specified. The ideal size for circle detection in this study ranged from 5 to 10 pixels, with only bright circles selected. It is important to consider the sensitivity of the identified circle. Lowering the sensitivity increases the detection of false circles (noise). In this study, an optimal sensitivity value of 0.92 was found, resulting in the detection of ten defects in grayscale images and nine defects in RGB images. Defects at positions B1 and C1, which had the highest depth at 2.88 mm compared to the others, were particularly difficult to detect, especially in RGB images. Figure 30 displays the result of the circle segmentation technique, with blue circles indicating detected defects.

### 3.7. Tanimoto Criterion Result

The Tanimoto criterion was used to validate the optimization of parameters for detecting FBH defects in GFRP material on raw images and after image processing. Tanimoto provides a quantitative measure of similarity between two sets, which makes it suitable for comparing defect and non-defect regions and has been widely used to analyze the performance of detection defects [55,71].

Verification was performed on the output results obtained at 30 s of heating for each tested parameter. The results show that without optimization, the detection results varied from 0 to 0.9, depending on the type of parameter tested. Some of the TC results were high even though the parameters were not optimized, but the images were blurry and unclear. After optimization, the Tanimoto criterion increased to 0.91 with optimized parameters at low temperatures (16 °C to 18 °C) and room temperature (23 °C to 25 °C). In terms of the clarity of defects, the results with optimized parameters were much clearer. Detailed Tanimoto results for each parameter, with and without parameter optimization, are provided in Table 4.

The Tanimoto criterion was also tested on several image segmentation methods applied to the raw images captured using the LPT setup. The results in Table 5 revealed that the Tanimoto detection efficiency varied from 0.17 to 0.91 after image processing for the FBH hole defect of sample A. The circle detection segmentation method using RGB color and Canny edge detection were the most efficient image-processing methods for automatic defect detection, achieving an efficiency of 0.91. Histogram threshold segmentation showed the lowest efficiency at 0.17. The circle detection segmentation method achieved a Tanimoto criterion result of 0.82, but this method is suitable only for FBH defects.

In summary, using an enclosure can prevent background reflections from the surroundings or any heat source from interfering with the image-capturing process using an IR camera. Different emissivity colors other than black resulted in increased noise and uneven surface heating. Utilizing black tape with an emissivity near 1 enhances noise rejection and improves defect detection as a result. Heating profile factors, such as the heating duration and cooling period, also contribute to the detection of defects. Consequently, for longer-distance detection using the LPT method, the energy of the heating source and the chosen heating duration should be sufficient to allow heat to reach the surface of the testing sample and heat the sample evenly.

The results of the parameter optimization on FBH defects of sample A, captured using LPT thermography with a low-resolution and uncooled microbolometer IR camera, demonstrated the detection of all 11 defects. However, B1, with a depth of 2.88 mm and a diameter of 21 mm, and C1, with the same depth and a diameter of 14 mm, showed low observability. This is due to B1 and C1 having the highest depths, with depth/diameter ratios of 0.137 and 0.206, respectively, compared to other FBH defects on the sample. The maximum number of defects detected matched those using cooled and high-resolution IR, where a 2 mm FBH defect was detected using SHT, one of 2 mm was detected using LP, a 1 mm depth was detected using PT [44], a 3.5 mm depth was detected using SHT [54], and a 1.6 mm depth was detected using improved ECPT [72]. However, the depth/diameter ratio needs improvement. The Tanimoto criterion, a common method for evaluating the performance of thermographic detection and processing methods, incorporates true positive (TP), true negative (TN), and false negative (FN) counts. The detection of FBH defects in GFRP composites using the LPT method improved to a TC of 0.91 with the optimized parameters.

## 4. Conclusions

This study demonstrated the effectiveness of the Long Pulse Thermography (LPT) method, employing low-resolution and uncooled IR cameras, for the assessment of GFRP composite materials. Through the meticulous evaluation and optimization of several key parameters, we identified the optimal conditions for defect detection, significantly enhancing the accuracy and reliability of the LPT method.

The experimental results showed that controlling the environmental conditions and employing a closed setup with an optimized heating duration and distance greatly reduced noise and improved defect visibility. The application of black tape to increase surface emissivity proved essential in enhancing defect detection capabilities by minimizing background reflections and ensuring even heat distribution across the material surface. The integration of advanced image processing techniques, such as edge detection and circle segmentation, further improved the detection accuracy of FBH and impact defects. The Tanimoto criterion results highlighted the effectiveness of these techniques, with the Canny edge detection and circle detection methods achieving the highest detection efficiency. By optimizing the LPT setup and employing robust image processing algorithms, we successfully detected 91% of FBH defects in GFRP composites. This level of detection is comparable to, and in some cases exceeds, the performance of more sophisticated and costlier high-resolution IR camera systems.

The findings demonstrate the potential of low-cost, uncooled, low-resolution IR cameras to reliably and accurately detect defects on flat GFRP. This approach provides a cost-effective solution for small- and medium-sized industries seeking to implement efficient non-destructive testing (NDT) methods for composite material health monitoring. Future work will focus on further refining the parameter optimization and image processing techniques, as well as exploring the application of this method to other types of composite materials and defect scenarios. Additionally, the development of standardized guidelines for LPT thermography using uncooled and low-resolution IR cameras will be pursued to ensure consistent and reliable applications across various industries.

## Figures and Tables

**Table 1:** Defect size and thickness.

No. of Defects	Defect	Diameter (mm)	Depth (mm)
1	A1	26	1.96
2	A2	26	2.00
3	A3	26	1.18
4	A4	26	1.92
5	B1	21	2.88
6	B2	21	1.88
7	B3	21	1.00
8	B4	21	0.96
9	C1	14	2.88
10	C2	14	2.80
11	C3	14	1.88

**Table 2:** Important parameters selected for the LPT thermography method.

Parameter Testing
Environment condition and surrounding temperature
Internal enclosure color background reflection
Background reflection Material surface emissivity

**Table 3:** Important parameters with the optimized value and conditions.

Parameter	Optimized Value and Condition
Environment and temperature	Indoors at a low temperature (16 °C)
Surface emissivity	Black tape on material
Closed or open setup	Closes setup
Background color	Black

**Table 4:** Tanimoto criterion on raw images captured using the LPT method.

	Image	Surface Emissivity			Indoor/Outdoor		Enclosure		Internal Color Enclosure			Temperature	
TC		Black	No Tape	White	Indoor	Outdoors	Yes	No	Y	W	B	16–18 °C	23–25 °C
0.43	Blurry		x			x		x					
0.91	Blurry		x		x		x				x		x
0.81	Burry,		x		x		x					x	
0.70	Burry,		x		x		x				x	x	
0.50	Blurry		x		x		x		x			x	
0.50	Blurry		x		x		x			x		x	
0.90	Blurry	x			x			x					x
0.91	Clear, less noise	x			x		x				x		x
0.91	Clear, less noise	x			x		x				x	x	

**Table 5:** Tanimoto criterion on defect detection using image segmentation methods.

	Edge Detection (Sobel)	Edge Detection (Canny)	Histogram Threshold	Circle Detection (RGB)	Circle Using (Grayscale)
No. of right detected	11	9	6	11	11
No. of mis detected	3	2	5	2	1
No. of false detected	1	1	0	0	0
TC	0.64	0.91	0.17	0.82	0.91

## Data Availability

All data are presented in the article.
